# Site-Specific, Critical Threshold Barefoot Peak Plantar Pressure Associated with Diabetic Foot Ulcer History: A Novel Approach to Determine DFU Risk in the Clinical Setting

**DOI:** 10.3390/medicina58020166

**Published:** 2022-01-21

**Authors:** Caroline A. Abbott, Katie E. Chatwin, Satyan M. Rajbhandari, Kanwal M. John, Sushma Pabbineedi, Frank L. Bowling, Andrew J. M. Boulton, Neil D. Reeves

**Affiliations:** 1 Department of Life Sciences, Faculty of Science and Engineering, Manchester Metropolitan University, Manchester M1 5GD, UK; K.Chatwin@mmu.ac.uk (K.E.C.); KMJ-95@hotmail.co.uk (K.M.J.); sushma.pabbineedi@gmail.com (S.P.); N.Reeves@mmu.ac.uk (N.D.R.); 2 Institute of Sport, Manchester Metropolitan University, Manchester M1 7EL, UK; 3 Diabetes Centre, Chorley and South Ribble Hospital, Lancashire Teaching Hospitals NHS Foundation Trust, Chorley PR7 1PP, UK; Satyan.Rajbhandari@lthtr.nhs.uk; 4 School of Medicine, Division of Diabetes, Endocrinology and Gastroenterology, University of Manchester, Manchester Royal Infirmary, Manchester M13 9PL, UK; Frank.Bowling@manchester.ac.uk; 5 Manchester Diabetes Centre, Division of Diabetes, Endocrinology and Gastroenterology, University of Manchester, Manchester Royal Infirmary, Manchester M13 9PL, UK; andrew.j.boulton@manchester.ac.uk; 6 Diabetes Research Institute, University of Miami, Miami, FL 33136, USA

**Keywords:** diabetes, foot ulcer, critical threshold, PressureStat™, peak plantar pressure, pressure measurement device

## Abstract

*Background and Objectives:* Barefoot peak plantar pressures (PPPs) are elevated in diabetes patients with neuropathic foot ulcer (DFU) history; however, there is limited reported evidence for a causative link between high barefoot PPP and DFU risk. We aimed to determine, using a simple mat-based methodology, the site-specific, barefoot PPP critical threshold that will identify a plantar site with a previous DFU. *Materials and Methods:* In a cross-sectional study, barefoot, site-specific PPPs were measured with normal gait for patients with DFU history (*n* = 21) and healthy controls (*n* = 12), using a validated carbon footprint system. For each participant, PPP was recorded at twelve distinct plantar sites (1st–5th toes, 1st–5th metatarsal heads (MTHs), midfoot and heel), per right and left foot, resulting in the analysis of *n* = 504 distinct plantar sites in the diabetes group, and *n* = 288 sites in the control group. Receiver operator characteristic curve analysis determined the optimal critical threshold for sites with DFU history. *Results:* Median PPPs for the groups were: diabetes sites with DFU history (*n* = 32) = 5.0 (3.25–7.5) kg/cm^2^, diabetes sites without DFU history (*n* = 472) = 3.25 (2.0–5.0) kg/cm^2^, control sites (*n* = 288) = 2.0 (2.0–3.25) kg/cm^2^; (*p* < 0.0001). Diabetes sites with elevated PPP (>6 kg/cm^2^) were six times more likely to have had DFU than diabetes sites with PPP ≤ 6 kg/cm^2^ (OR = 6.4 (2.8–14.6, 95% CI), *p* < 0.0001). PPP > 4.1 kg/cm^2^ was determined as the optimal critical threshold for identifying DFU at a specific plantar site, with sensitivity/specificity = 100%/79% at midfoot; 80%/65% at 5th metatarsal head; 73%/62% at combined midfoot/metatarsal head areas. *Conclusions:* We have demonstrated, for the first time, a strong, site-specific relationship between elevated barefoot PPP and previous DFU. We have determined a critical, highly-sensitive, barefoot PPP threshold value of >4.1 kg/cm^2^, which may be easily used to identify sites of previous DFU occurrence and, therefore, increased risk of re-ulceration. This site-specific approach may have implications for how high PPPs should be investigated in future trials.

## 1. Introduction

The aetiology of plantar foot ulceration for people with diabetes is underpinned by the presence of peripheral neuropathy, leading to a loss of protective sensation, deformities in the bony architecture of the foot and soft tissue changes, culminating in elevated weight-bearing plantar pressures and tissue breakdown [1,2]. The consensus in the literature is that barefoot peak plantar pressure (PPP) is considerably elevated in diabetes patients with neuropathy and foot ulcer (DFU) history [3,4,5], likely reflecting the foot deformities otherwise offset by therapeutic footwear/orthoses. Despite this, there is surprisingly little compelling evidence for a strong causative link between high PPP and incident DFU. Whereas neuropathy and DFU history are consistently reported as the strongest clinical predictors of new DFU [6,7,8], only a few prospective studies have demonstrated any predictive power of barefoot PPP for DFU [9,10,11,12], with only moderate correlations reported between locations of DFUs and PPP. This illustrates the complexities of abnormal plantar pressure distributions on the development of DFUs [13].

Although current guidelines advise diabetes patients with neuropathy to use adequately off-loading therapeutic footwear on a daily basis to reduce elevated plantar pressures and DFU risk [8], the lifetime risk of DFU remains high at up to 25% [14,15], despite such interventions. It is intuitive to consider that any non-adherence to wearing therapeutic footwear at home, i.e., walking barefoot during daily activities, would create an increased risk of new plantar tissue damage and, subsequently, contribute to the high lifetime risk of DFU. Yet, inconsistent results from prospective studies exploring the relationship between barefoot PPP and new DFU does not reflect this hypothesis. It could be considered that ‘whole plantar area’, barefoot PPP assessments undertaken in previous DFU risk studies, rather than adopting a ‘site-specific’ plantar area approach, may have masked the predictive power of PPP for DFU risk.

There are, to date, no DFU risk reports of the distribution of barefoot PPP at distinct plantar sites, the site-specific relationship between barefoot PPP with DFU or barefoot PPP critical thresholds above which DFU develop. Maintaining PPP below a threshold of 200 kPa has been recommended as the target in-shoe pressure threshold in footwear prescription for patients with prior ulcers [16]. This is not, however, a translatable threshold for barefoot walking, as barefoot PPPs may be up to four times higher than in-shoe PPP due largely to the exposure of bony prominences caused by foot deformities [12]. 

Plantar DFU location is discrete and site-specific, occurring predominantly at forefoot sites—often at the great toe and metatarsal heads (MTHs)—with mid-foot sites commonly affected in Charcot patients [17,18,19]. We suggest that critical high-pressure loading at discrete, specific plantar sites during barefoot gait may be more strongly associated with sites of previous plantar DFU (and thus, future risk of DFU) than elevated PPPs measured across the entire area of the ‘at risk’ foot.

In a cross-sectional analysis of a cohort of ‘high-risk’ people with diabetes with a previous history of plantar DFU, we aimed to: (1) determine the prevalence and distribution of individual plantar sites with critically high barefoot PPP; (2) determine whether previously ulcerated sites remain under high PPP (and therefore explain the high-risk of DFU recurrence); (3) determine a site-specific, critical threshold of barefoot PPP for DFU history, in order to help the footcare team identify and target occasional ‘high-risk’ plantar areas.

## 2. Materials and Methods

### 2.1. Study Design

This study was a cross-sectional cohort study of a sub-cohort of 21 people with diabetes with a previous history of plantar DFU, participating in a randomised, proof-of-concept trial of an intelligent insole system for DFU prevention [20]. Participants were recruited from March 2014 to December 2016.

The study was approved by local research ethics committees on 24 October 2013 and other relevant governance bodies in the UK (clinical trial registration number: ISRCTN05585501; NHS REC reference number: 13/NW/0649).

### 2.2. Participants

Participants with diabetes (*n* = 21), who had been previously referred by their general practitioner to a secondary care, multi-disciplinary outpatient diabetes foot clinic in central Manchester, UK, for their foot ulcer assessment and management, were recruited to the study. Inclusion criteria were age ≥ 18 years, Type 1 or Type 2 diabetes, history of previous ulceration on the weight-bearing surfaces of the foot, presence of diabetic polyneuropathy as defined by any loss of sensation, ability to walk independently for 30 steps. Main exclusion criteria included: active foot ulceration, severe vascular disease, lower limb amputation above the level of the ankle, BMI > 40 kg/m^2^ [20]. All patients provided written consent before enrolment. Healthy, age-matched control participants (*n* = 12) who had never received a clinical diagnosis of diabetes, and who were staff at Manchester Royal Infirmary, were also consented and recruited into the study.

### 2.3. Assessments

Demographic, medical and social variables were obtained for participants with diabetes. Details of historical plantar ulcers were documented from podiatry/medical notes. A detailed foot examination identified any amputations and foot deformities. Sensory loss for any of the modalities of the modified neuropathy disability score (NDS) classified patients with neuropathy. Callus severity was scored at 12 distinct plantar sites per foot, as previously described [20].

Barefoot peak plantar pressures during normal gait were measured using the simple, validated carbon footprint system, PressureStat™ (Arche Healthcare Inc, New York, NY, USA), formerly known as Podotrack (Visual Footcare Technologies Inc, New York, NY, USA) [21]. At the study visit, participants with diabetes were asked to walk barefoot on a carbon footprint mat from heel-strike to toe-off, during the first footstep, completing one full gait cycle only, while maintaining as natural a gait as possible, under the supervision of a podiatrist. In addition, *n* = 12 healthy, age-matched controls without diabetes underwent the same procedure.

### 2.4. Pressure Analysis

Following data collection, three researchers trained in PressureStat™ assessment [21] independently reviewed all carbon footprints, blinded to participant or group identity, as previously described [21]. Each researcher quantified the PPP at each of twelve distinct plantar sites per footprint (1st–5th toes, 1st–5th metatarsal heads, midfoot and heel) by visually comparing the greyness of the carbon footprint mat with a specially designed calibration card containing seven different gradings of grey, each characteristic of a specific range of pressure measurements: 0–0.5 kg/cm² (0–49 kPa); 0.5–1.5 kg/cm² (49–147 kPa); 1.5–2.5 kg/cm² (147–245 kPa); 2.5–4 kg/cm² (245–392 kPa); 4–6 kg/cm² (392–588 kPa); 6–9 kg/cm² (588–883 kPa); 9–15+ kg/cm² (883–1471 kPa). During quantification, each researcher positioned the calibration card, at arm’s length, next to each of the footprint areas, using standardised background lighting. Within each of the twelve distinct plantar sites per footprint, each researcher visually selected the greyest point, no matter how small the greyest point was within that area. The equivalent, closest grade of grey on the calibration card to that area was chosen, and the corresponding PPP range was then recorded.

The intraclass correlation coefficient (ICC) for inter-observer reliability for PPP recordings was high for both the control group (0.752; 95% CI: 0.697–0.798; *p* < 0.001) and the diabetes group (0.885; 95% CI: 0.860–0.90; *p* < 0.001); therefore, one analyser’s results alone was randomly selected for all further data analyses. Focal plantar pressure of ≥ 6 kg/cm^2^ (equivalent to ≥ 588 kPa) was used as the initial standard definition for critically ‘high’ pressure during barefoot walking [4].

At monthly longitudinal follow-up visits, participants’ feet were examined to identify any new or recurrent plantar ulcers, defined as a full-thickness loss of epidermis and dermis or involvement of deeper structures, to at least Texas classification Stage 1 [22], on the weight-bearing surface of the foot.

### 2.5. Statistical Methods

Descriptive statistics of continuous variables were reported as means with SDs, or medians with IQR and 95% CIs (for categorical variables, proportions and frequencies were given). All data were checked for normality of distribution by examining histograms and comparison of mean/median values. Baseline data were compared between groups of participants with diabetes versus healthy controls using an independent Student’s *t*-test for parametric data and Fisher’s exact test for proportions.

For the inter-observer reliability analysis, a two-way mixed-effects consistency multiple-observers/measurements ICC model was used.

The frequency distribution of PPP recordings for (a) all control sites (*n* = 288), (b) all diabetes sites without DFU history (*n* = 472), and (c) all diabetes sites with DFU history (*n* = 32) were compared between the groups using a Kruskal-Wallis one-way ANOVA.

For each individual plantar site analysed, mean PPP recordings for (a) control sites, (b) diabetes sites without DFU history and (c) diabetes sites with DFU history were compared between groups using a one-way ANOVA and subsequent post-hoc Scheffé test.

Logistic regression analysis models were constructed to assess the predictive value of PPP cut-off values for identifying DFU history for diabetes plantar sites only (odds ratios equate to relative risks when outcomes are < 10%). Receiver operator characteristic (ROC) curves were used to generate performance thresholds for PPP and examine the sensitivity/specificity of these thresholds at different plantar sites. All individual plantar sites were considered to be independent of sites on the same or other foot for each participant, based on evidence from prior research [20] and the present study. Statistical analysis was conducted using SPSS Version 25 (IBM, Chicago, IL, USA).

## 3. Results

The baseline characteristics of those with diabetes (*n* = 21) and the age-matched healthy controls (*n* = 12) are in Table 1. There were 32 previous plantar DFUs recorded. Previous plantar DFUs were predominantly located at MTHs 1–5 (*n* = 12, (38%)) and the hallux (*n* = 11, (34%)), with other sites being at the toes 2–5 (*n* = 5, (16%)), midfoot (*n* = 3, (9%)) and heel (*n* = 1, (3%)). The mean duration since the previous ulcers had healed was 5.5 ± 3.9 years.

In the diabetes cohort, PPP was assessed at 504 individual plantar sites (12 plantar sites × 2 feet × 21 patients = 504). The total number of sites with ‘high’ PPP (≥6 kg/cm^2^) was 41, i.e., just 8.1% of all plantar sites assessed. The total number of sites with PPP ≥9 kg/cm^2^ (‘very high’ PPP) was 5/504, i.e., <1% of all plantar sites assessed. In the control group, the total number of plantar sites assessed for PPP was 288 (12 plantar sites × 2 feet × 12 patients = 288). The number of sites with PPP ≥6 kg/cm^2^ (‘high’ PPP) was 0/288 (0%).

Analysis of the 42 individual feet in the diabetes cohort showed that 45% feet (*n* = 19) had zero ‘high’ PPP sites (≥6 kg/cm^2^), 36% (*n* = 15) had 1 high PPP site, and 19% (*n* = 8) had two or more high PPP sites. There was an average of just 1 high PPP site per foot.

Mean PPP levels at specific plantar areas in all individual diabetes feet (*n* = 42) were highest at the 5th MTH (4.6 ± 2.5 kg/cm^2^), 2nd MTH (4.3 ± 2.2 kg/cm^2^), great toe (4.3 ± 1.6 kg/cm^2^), 1st MTH (4.2 ± 2.0 kg/cm^2^) and 4th MTH (4.1 ± 1.5 kg/cm^2^). All other plantar sites had mean PPP < 4 kg/cm^2^(data not shown). Mean PPP at the great toe was significantly higher in feet with general toe deformities (claw/hammer toes) (*n* = 22 feet; great toe PPP = 5.1 ± 1.3 kg/cm^2^), than feet without toe deformities (*n* = 19 feet; great toe PPP = 3.3 ± 1.4 kg/cm^2^, *p* < 0.0001).

### 3.1. Cross-Sectional Analysis of PPP and DFU Risk

**Figure 1:**
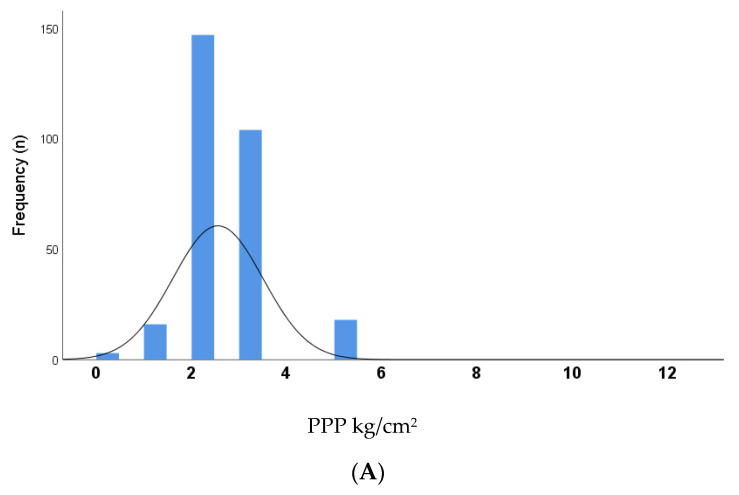

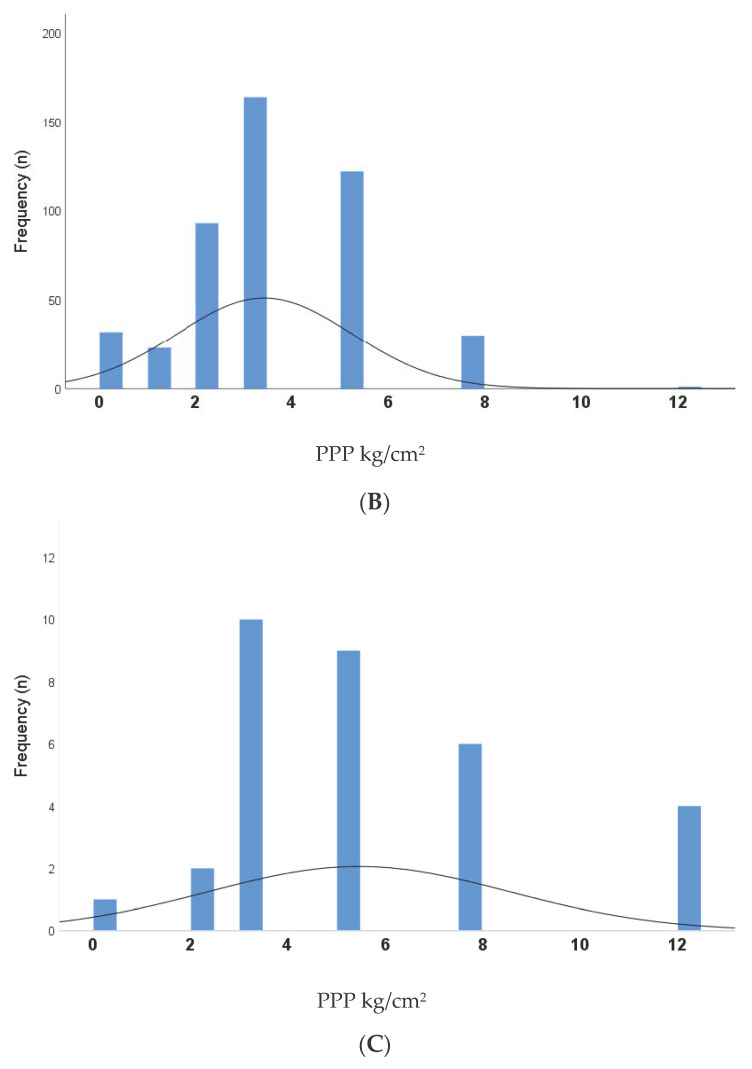
Distribution curves for PPP (peak plantar pressure) values at specific plantar sites (*n* = 792) for all participants (*n* = 33); split into 3 sub-groups for analysis: (**A**) PPP values at healthy control sites (*n* = 288); (**B**) PPP values at diabetes sites without DFU history (*n* = 472); (**C**) PPP values at diabetes sites with DFU history (*n* = 32). Median (IQR) PPP for the groups were: a) control sites = 2 (2–3.25) kg/cm^2^; diabetes sites without DFU history = 3.25 (2–5) kg/cm^2^; diabetes sites with DFU history = 5 (3.25–7.5) kg/cm^2^ respectively and were significantly different (*p* < 0.0001).

Figure 1 shows the frequency distribution curves for all PPP values (*n* = 792) recorded at specific plantar sites for all (*n* = 33) study participants, split into 3 sub-groups for analysis: (A) healthy control sites (*n* = 288); (B) diabetes sites without DFU history (*n* = 472); (C) diabetes sites with DFU history (*n* = 32). Median (IQR) PPP for the groups were: healthy control sites = 2 kg/cm^2^ (2–3.25); diabetes sites without DFU history = 3.25 kg/cm^2^ (2–5); diabetes sites with DFU history = 5 kg/cm^2^ (3.25–7.5), and all 3 groups were significantly different (*p* < 0.0001). Curves for control sites and diabetes sites without DFU history were normally distributed/moderately skewed (skewness = 0.69 and 0.53, respectively); whereas the curve for diabetes sites with DFU history was moderately/highly skewed towards the right (skewness = 0.97).

**Figure 2:**
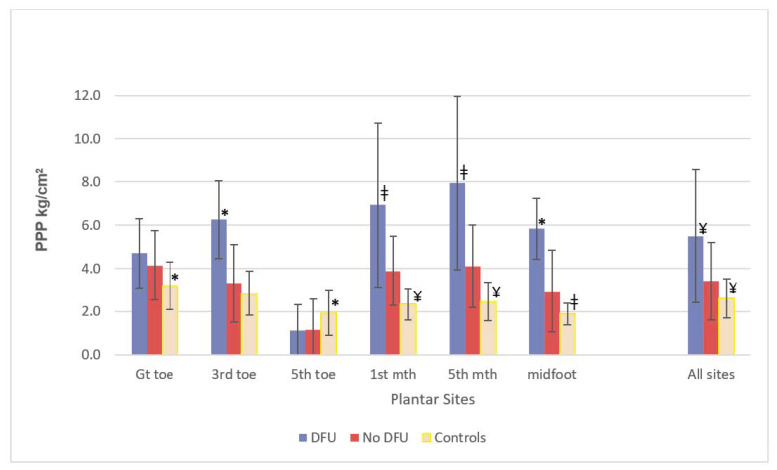
Comparison of mean PPP levels between different groups of plantar sites: diabetes sites with DFU history (total sites, *n* = 32); diabetes sites with no DFU history (total sites, *n* = 472); healthy control sites (total sites, *n* = 288). * *p* < 0.05’, ‡ *p* < 0.01, ¥ *p* < 0.0001, Group vs. No DFU group.

A comparison of mean PPP levels between the three groups of plantar sites (healthy control sites (*n* = 288), diabetes sites without DFU history (*n* = 472) and diabetes sites with DFU history (*n* = 32), is given in Figure 2. Mean PPP was significantly greater at all plantar sites with a DFU history compared to all plantar sites with no DFU history (*p* < 0.0001). Furthermore, specific, individual sites with DFU history (5th MTH, 1st MTH and midfoot sites) had significantly higher PPP (8.0 ± 4.0 kg/cm^2^, 6.9 ± 3.8 kg/cm^2^ and 5.8 ± 1.4 kg/cm^2^, respectively) than their equivalent sites without DFU history (4.1 ± 1.9 kg/cm^2^, 3.9 ± 1.6 kg/cm^2^ and 2.9 ± 1.9 kg/cm^2^, respectively; *p* < 0.05). In contrast, there was no difference at the great toe for PPP when comparing DFU history sites vs. no DFU history sites; furthermore, across all conditions, PPPs were generally lower at the hallux than other plantar sites. All healthy control sites had lower mean PPP compared to all diabetes group sites without a history of DFU (*p* < 0.0001). Similarly, the control group mean PPP was consistently lower at each individual site between these two groups: at the great toe, 1st–5th MTHs, midfoot and heel (*p* < 0.05).

For all diabetes sites (with and without a DFU history), a logistic regression analysis model was constructed to assess the predictive value of site-specific ‘high’ (≥ 6 kg/cm^2^) PPP for identifying DFU history. The model was DFU site history vs. no DFU site history, using a PPP threshold of ≥ 6 kg/cm^2^ at that site (n = 504 sites included). The odds ratio for this model was 6.4 (2.8–14.6, 95% CI), *p* < 0.0001, indicating that any plantar sites with PPP ≥ 6 kg/cm^2^ were six times more likely to have a history of DFU than sites with PPP below this threshold. This ‘site-specific’ model showed a much stronger and predictive OR compared to an alternative ‘whole foot’ model, i.e., DFU site history vs. no DFU site history, with high PPP threshold of (≥ 6 kg/cm^2^) at any plantar site on the foot (*n* = 504). In the latter ‘whole foot’ model, the OR showed no association between DFU site history and high whole-foot PPP (OR = 1.4 (0.67–2.93), *p* = 0.37).

#### ROC Curves and Critical Threshold for DFU History

**Figure 3:**
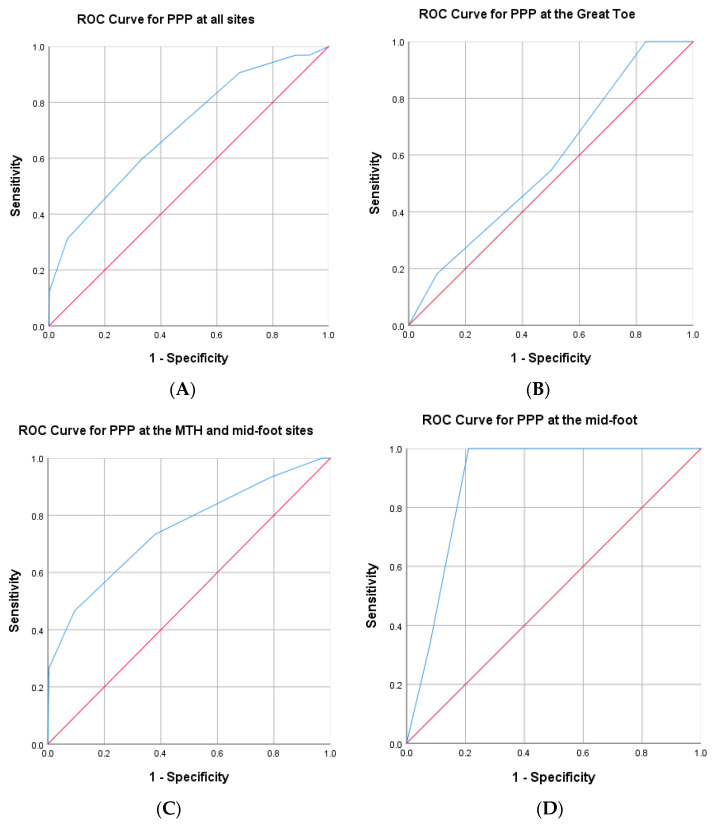
Receiver operating characteristic (ROC) curves for peak plantar pressure at (**A**) all plantar sites (*n* = 504 sites); (**B**) great toe only (*n* = 42 sites); (**C**) combined metatarsal and mid-foot sites (*n* = 252 sites); (**D**) mid-foot only (*n* = 42 sites). The ROC curves demonstrate the sensitivity and specificity for identifying plantar areas with a history of DFU at a threshold of 4.1 kg/cm^2^. A curve closer to the top left-hand corner indicates a more sensitive and specific test. The diagonal (45°) line serves as a baseline for the area under the curve. (**A**) PPP threshold of 4.1 kg/cm^2^ at any plantar site has 59% sensitivity and 67% specificity for predicting DFU history. Area under the curve = 0.70 (95% CI: 0.60–0.80; *p* < 0.0001), fair/good diagnostic ability. (**B**) Area under the curve = 0.58 (95% CI: 0.39–0.77, *p* = 0.44). There was no diagnostic ability of PPP at the great toe for predicting DFU history. (**C**) PPP threshold of 4.1 kg/cm^2^ at the metatarsal heads and mid-foot had 73% sensitivity and 62% specificity for predicting DFU history. Area under the curve = 0.75 (95% CI: 0.61–0.90; *p* < 0.001), very good diagnostic ability. (**D**) PPP threshold of 4.1 kg/cm^2^ at the mid-foot alone had 100% sensitivity and 79% specificity for predicting DFU history. Area under the curve = 0.89 (95% CI: 0.78–1.00; *p* = 0.026), excellent diagnostic ability.

Figure 3 shows the ROC curves for peak plantar pressures at: (A) all plantar sites (*n* = 504 sites); (B) great toe only (*n* = 42 sites); (C) combined metatarsal and mid-foot sites (*n* = 252 sites); (D) mid-foot only (*n* = 42 sites). The area under the ROC curves (A), (C) and (D) were large and significantly different from a value of 0.5 (Figure 3), and curve coordinates showed that the PPP critical threshold of 4.1 kg/cm^2^ (402 kPa) was the most reliable prediction method of DFU history at a specific plantar site and was significantly better than the established > 6 kg/cm^2^ threshold (Figure 3). The 4.1 kg/cm^2^ PPP threshold had a fair diagnostic ability when tested at any plantar site (Figure 3A), with a sensitivity of 0.59 and specificity of 0.67. This means that the 4.1 kg/cm^2^ PPP threshold correctly classified 59% of all plantar sites that had a previous DFU and correctly classified 67% of all plantar sites that had no previous DFU. When testing at the metatarsal heads and midfoot only (Figure 3C), the diagnostic ability of the 4.1 kg/cm^2^ threshold was very good, with the test sensitivity rising to 73%. Testing at distinct, individual plantar sites using the 4.1 kg/cm^2^ threshold for PPP generally showed much better sensitivities and specificities than for whole foot (any site). At midfoot alone (Figure 3D), sensitivity was maximum at 100%, and specificity was high at 79%. For the 5th MTH alone (area under the ROC curve = 0.81, 95% CI: 0.60–1.0, *p* = 0.026) sensitivity was 80% and specificity was 65%. Conversely, however, the ROC curve for PPP at the great toe site was not significantly different from 0.5 and had no diagnostic ability.

## 4. Discussion

This study has demonstrated, for the first time, a strong, site-specific relationship between ‘elevated’ barefoot PPP and DFU history for ‘high-risk’ patients. Furthermore, we found surprisingly low numbers of discrete areas of high pressure across the plantar surface of ‘high-risk’ feet (less than one area per foot, on average), despite the general current assumption that ‘high-risk’ feet are at risk of re-ulceration across the plantar surface because of generally higher PPPs. We have also determined a critical barefoot peak plantar pressure threshold value of >4.1 kg/cm^2^ (>402 kPa), with optimal sensitivity for predicting these ‘high-risk’ plantar sites. The methodology for this, using a single-use carbon footprint mat, could be adopted very easily into the clinical setting.

Our evidence was (a) plantar sites with established ‘high’ barefoot PPP (>6 kg/cm^2^ (i.e., >588 kPa)) were six times more likely to have a history of DFU than sites with barefoot PPP below this threshold; (b) however, there was no relationship between DFU sites and ‘high’ barefoot PPP (>6 kg/cm^2^) recorded more generally across the plantar surface of the foot; (c) ‘high’ barefoot PPP (>6 kg/cm^2^) sites were infrequent across the whole plantar surface, averaging just one ‘high’ PPP site per foot; (d) ROC curve coordinates showed that PPP critical threshold of 4.1 kg/cm^2^ (402 kPa) was the most reliable prediction method of DFU history at any plantar site, and significantly better than the established >6 kg/cm^2^ threshold; (e) sensitivity (the ability to correctly identify the DFU site) of the 4.1 kg/cm^2^ threshold was highest at the metatarsal heads and midfoot sites (73%) and peaked at 100% at midfoot alone (however, there was no diagnostic capability at the great toe). All of this evidence indicates that there is a very strong relationship between barefoot PPP and DFU history occurring at discrete plantar sites per foot. These sites are very easily identifiable in the clinical setting using a single-use carbon footprint mat, with a highly sensitive threshold for capturing the DFU risk at the metatarsal heads and midfoot regions, enabling a simple, cost-effective, site-specific, targeted approach for off-loading foot management.

The majority of previous studies of barefoot PPP and DFU risk have examined the effect of PPP averaged across the whole plantar area, at best, forefoot:rearfoot distribution of mean barefoot PPPs [3,4,5,10,12,23,24], in contrast to our unique, site-specific approach. For the first time, we have teased out (from 12 distinct plantar regions) that the DFU sites most strongly associated with elevated PPP were the 1st MTH, 5th MTH and midfoot. In our high-risk cohort, DFU history was most prevalent at the metatarsal heads (38%), as described similarly elsewhere [3,12,23,24], and was closely related to elevated PPP at these forefoot sites. Interestingly, however, we found that the great toe sites, despite having elevated PPPs and constituting one-third of all diabetes foot plantar areas with DFU history, showed no difference in mean PPP when comparing groups ‘DFU history at great toe site’ with ‘no DFU history at great toe site’ (Figure 2); this was atypical of all other plantar areas. This lack of a relationship between elevated barefoot PPP and DFU risk at the great toe, unlike all other forefoot/midfoot sites, is a novel finding. Additionally, PPPs at the great toe with DFU history were lower than all other plantar areas with DFU history (Figure 2). The current literature describes that the greatly increased DFU risk at the neuropathic great toe (such as for our patients) results from the generally increased prevalence of hallux limitus (great toe stiffness, i.e., reduced ability of metatarsophalangeal joint extension), potentially predisposing the great toe to increased plantar pressure during gait [25]. We showed that elevated PPP at the great toe has no relationship with the high prevalence of DFU at the great toe, whereas elevated PPP is strongly associated with DFU at all other plantar sites. Other factors, such as increased plantar shear pressures, are also considered to have an important role in plantar DFU pathology [26]. The high DFU rate at the great toe could at least be partly explained, therefore, by elevated shear pressure, rather than predominantly elevated vertical PPP. These findings reassert the importance of the site-specific relationship between DFU risk and plantar pressure and probably explains the weak ‘whole-foot’ PPP relationship with DFU risk.

One other DFU risk study has separated pressure analysis at previous DFU sites into regions instead of combining all DFU and pressure data [17]; however, this study used in-shoe, not barefoot, PPP analyses. Similar to our findings, the authors showed that both the hallux and metatarsals had the highest DFU rate, yet only higher baseline PPP at metatarsal sites was significantly associated with greater DFU risk, whereas consistent with our data, this relationship was non-significant at the hallux. Their data, therefore, indicated a location-specific relationship at the metatarsals, but not the hallux, similar to our findings.

The very strong association of DFU history when PPP is above the 4.1 kg/cm^2^ threshold at the midfoot here also supports previous findings of DFU history causing increased loading under the midfoot during barefoot gait, with a general increase in variability of plantar pressure [27]. Although PPPs are generally highest at the forefoot of Charcot feet [28], we show here that breaching even the relatively low critical threshold at the midfoot is still associated with DFU history at this site. Our critical threshold is lower than that previously reported (>6 kg/cm^2^ (588kPa)) as ‘high-risk’ for barefoot walking [4]; however, in the previous cross-sectional study of patients with diabetes, PPPs were obtained for the entire foot without regard for the specific location and their threshold was chosen only as it lies one SD above the mean of healthy subjects. Indeed, our critical threshold is identical to that proposed (but not tested) by Stess and colleagues for forefoot ulceration in neuropathic diabetic patients (>40 N/cm^2^ (i.e., >4.1 kg/cm^2^)) [3].

The barefoot PPP range for our cohort is highly comparable to barefoot PPP ranges observed in similar populations [12,16,29]. Waaijman et al. found that mean barefoot PPP at previous DFU sites, which subsequently re-ulcerated, was 849 (±375) kPa (SD), which equates to the 6–9 kg/cm^2^ range for PressureStat™. Of note, their model examining ‘all DFU recurrences per foot’ showed that barefoot PPP at ‘plantar foot distal to the heel’ was not independently significant in the multivariate model; conversely, PPP was independently significant in their site-specific model. This is important data, highlighting a site-specific relationship between PPP and DFU; unfortunately, information regarding actual locations on the plantar foot was not included in their study. Elsewhere, mean PPP at the ‘forefoot’ during barefoot walking in patients with previous DFU (mean = 566 kPa (range =107–1192 kPa) [16] was very similar to our findings (490 kPa (24.5–1177 kPa (equivalent values)); furthermore, barefoot PPP ≥ 450 kPa (i.e., 4.6 kg/cm^2^) in diabetic patients with neuropathy has previously been considered as a ‘region of interest’ [29]. Our findings that barefoot PPP threshold ≥ 4.1 kg/cm^2^ provides optimal sensitivity and specificity for identifying risk of DFU site validate this proposed cut-off value.

The above studies, in which in-shoe plantar pressures are reduced compared to equivalent barefoot pressures (up to a 4-fold reduction) [12,16,29], support current guidelines which recommend that diabetic patients with neuropathy should always wear footwear during daily activities to reduce pressure and risk of DFU. Indeed, in-shoe peak pressure < 200 kPa with ‘good adherence’ appears to protect against DFU recurrence, although footwear interventions are often described as being associated with poor adherence, limiting their effectiveness in preventing DFU [30]. Indeed, barefoot analysis enables an understanding of ‘base-level’ plantar pressure loading when patients are not wearing their therapeutic/off-the-shelf footwear, i.e., when patients are walking barefoot at home during daily activities. Our data suggest that the role of barefoot PPP in the risk of DFU may be stronger than the literature currently suggests, perhaps becoming more relevant during periods of poor adherence to therapeutic footwear, even for patients who are usually ‘good’ compliers. Any periods walking barefoot are possibly key periods in the aetiology of DFU recurrence.

The number of discrete areas of high pressure across the plantar surface of ‘high-risk’ feet were scarce (less than one area per foot, on average), despite the general current assumption that ‘high-risk’ feet are at risk of re-ulceration across the plantar surface because of generally higher PPPs. This novel finding has been enabled by the ability to capture an instant ‘snapshot’ plantar image of all barefoot PPPs using the carbon mat PressureStat™ system.

The PressureStat™ system used for our analyses is an inexpensive, validated, highly sensitive, quick and simple alternative device for measuring barefoot PPP foot pressures in the clinical setting [21], contrasting with most previous studies, which have used more expensive pressure platforms, restricted to laboratories [5,9,11,23]. The subjective nature of using a visual scale for analysis, as opposed to using an automated system, could be considered a methodology limitation; however, the excellent inter-observer reliability following operator training, plus peer-reviewed validation of this system against lab-based pressure measurement systems, counteracts this. A general disadvantage of barefoot analysis per se is the potential increased risk to the insensate neuropathic foot as the patient walks over the platform/PressureStat™ mat. In a strictly controlled, clinical environment, however, this methodology should be safe. In our study, we assessed the first footstep PressureStat™ for each foot, thus minimising the time spent barefoot for each patient, also demonstrating that steady-state gait velocity is not a necessity for PPP analysis. There are other limitations of our study, which may impact our interpretation of the results, including a lack of a control group of diabetic patients without a history of DFU, the presence of which would have provided further plantar pressure distribution data. Furthermore, it is possible that the lower weight and higher proportion of females in our healthy controls compared with the diabetes group, plus differences in foot deformities/callus severity (not measured in our healthy controls), may partly explain the lower PPPs found in the control group. As 95% of our diabetes group was male, our results also may not be generalisable to entire diabetes ‘high-risk’ population. We are aware that a prospective study (rather than cross-sectional) would have been the optimal design to demonstrate the predictive relationship between PPP and the risk of DFU recurrence. Such follow-up studies, however, are relatively rare as DFU incidence, even in high-risk patients, is often too low within the study timeframe to provide any meaningful data. In our present study (data not shown), only 3/21 participants with diabetes went on to develop a plantar DFU after 24.3 (23.2–46.7) weeks (median (IQR)) during an 18-month prospective follow-up. An exploratory logistic regression analysis model was constructed to assess the predictive value of the highest PPP category (9–15+ kg/cm^2^) for identifying new DFU. The model indicated that any plantar sites with PPP > 9 kg/cm^2^ were 60-fold more likely to develop a new DFU than sites with PPP below this critical threshold (*p* < 0.0001); however, because of the low number of events, the effect estimate was very large, and the model was considered unstable. Predictive analyses of this kind may prove to be successful with a larger participant sample size.

We recommend the use of this inexpensive (approximately $2 per footprint), robust screening tool in the annual review clinic, ideally at the point when patients’ shoes and socks have already been removed for foot examination and neuropathy screening, to capture evidence of high-risk barefoot plantar pressures. The carbon footprint provides an immediate visual image for the patient and physician to view and discuss together, acting as an educational tool for the patient and enabling the physician to immediately target specific, problem plantar areas for off-loading therapy. Furthermore, as we have shown for the first time, areas of high pressure on the ‘at-risk’ foot are very scarce; we suggest that this may have implications for how high PPPs should be investigated in future clinical trials.

## 5. Conclusions

To conclude, we have determined and validated a robust, highly sensitive, critical plantar pressure threshold value of >4.1 kg/cm^2^ to identify specific plantar sites of previous DFU occurrence, using an inexpensive, simple carbon footprint screening tool. We can recommend the use of this threshold value for ‘high-risk’ patients in daily clinical practice (rather than just for clinical research) to identify which individual plantar sites are at higher risk of DFU recurrence, enabling immediate, targeted DFU prevention strategies.

## Figures and Tables

**Table 1:** Baseline characteristics of participants. Results given as number (%) or mean (SD). Foot deformity score comprised a total of 6 abnormal scores (1) for each of the following per foot: hammer or claw toes, prominent metatarsal heads, small muscle wasting, bony prominences, Charcot, limited joint mobility was determined by assessing range of motion in the finger joints during the prayer sign. Callus severity score—callus severity was assessed at 12 distinct plantar sites per foot (first–fifth toes, first–fifth metatarsal heads, midfoot and heel). Scores were no callus (zero), mild callus (1), medium callus (2) and severe callus (3). Total callus severity scores were recorded for each foot at baseline (Abbott et al., 2019) [20].

Characteristics	Diabetes Group (*n* = 21)	Control Group (*n* = 12)
Age (years)	60.7 ± 9∙5	58.5 ± 8.3
Male gender, *n* (%)	20 (95.2%)	4 (33.3%)
Type 2 diabetes, *n* (%)	11 (52.4%)	
Diabetes duration (years)	28.8 ± 13.4	
HbA_1c_ (mmol/mol) (%)	64.7 ± 10.4 8.1 ± 3.1	
Ethnicity, *n* (%):		
White British/Other White	17 (81.0%)	10 (83.3%)
South Asian	2 (9.6%)	1 (8.3%)
Black/Mixed race	2 (9.6%)	1 (8.3%)
Weight (kg)	97.9 ± 22.2	71.9 ± 11.9
BMI (kg/m^2^)	30.9 ± 6.7	26.2 ± 3.8
Neuropathy Disability Score (NDS):	8.3 ± 2.1	
Previous toe amputations		
Hallux	1 (4.8%)	
2nd—5th toe	1 (4.8%)	
Previous DFU on left foot:		
Hallux	6 (28.6%)	
2nd—5th toe	2 (9.5%)	
1st—5th metatarsal heads	6 (28.6%)	
Midfoot	1 (4.8%)	
Heel	1 (4.8%)	
Previous DFU on right foot:		
Hallux	5 (23.8%)	
2nd—5th toe	3 (14.3%)	
1st—5th metatarsal heads	6 (28.6%)	
Midfoot	2 (9.5%)	
Heel	0 (0.0%)	
Claw/hammer toes—Left	13 (61.9%)	
Claw/hammer toes—Right	10 (47.6%)	
Prominent metatarsal heads—Left	10 (47.6%)	
Prominent metatarsal heads—Right	11 (52.4%)	
Charcot foot—Left	5 (25.0%)	
Charcot foot—Right	2 (9.5%)	
Limited Joint Mobility—Left	11 (52.4%)	
Limited Joint Mobility—Right	11 (52.4%)	
Foot Deformity Score—Left	2.5 ± 1.6	
Foot deformity score—Right	2.3 ± 1.5	
Callus severity score—Left	2.6 ± 3.1	
Callus severity score—Right	1.6 ± 2.3	

DFU: diabetic foot ulcer.

## Data Availability

The datasets generated during and/or analysed during the current study are available from the corresponding author on reasonable request.

## Abbreviations

PPP	peak plantar pressure
DFU	diabetic foot ulcer
MTH	metatarsal head
NDS	neuropathy disability score
ICC	intraclass correlation co-efficient
ROC	receiver operator characteristic
